# Retinoblastoma: Teacher of Cancer Biology and Medicine

**DOI:** 10.1371/journal.pmed.0020349

**Published:** 2005-10-25

**Authors:** Alfred Knudson

## Abstract

Retinoblastoma attracts the interest of oncologists as well as cancer biologists. Knudson discusses why retinoblastoma has been a model for guiding our understanding of cancer genes.

Despite its low incidence, retinoblastoma has attracted the great interest of not just pediatric oncologists, but also cancer biologists and oncologists who treat cancer in adults. Retinoblastoma reminds us that rare conditions with a hereditary component may be rare for a reason—the genes associated with them are important biologically. It is hardly surprising then that retinoblastoma continues to be a subject of interest to a large group of clinicians and scientists, as described by Dyer and colleagues in this issue of *PLoS Medicine* [1].

## A Success Story

For the pediatric oncologist, the treatment of retinoblastoma has been one of the success stories of the past century, with a cure rate in the United States of 95% [2,3]. The clinicians' concerns are now those children who do not survive, those who lose one or both eyes, and those who sustain delayed effects, including late death from a second cancer.

For cancer biologists, including myself, retinoblastoma has been a model for guiding our understanding of cancer genes. For me, it was a simplifying condition to study: a child could inherit a mutated gene that could produce a cancer even in a newborn, so such a gene must surely be important for cancer research. Indeed, the retinoblastoma gene *RB1* was the first hereditary cancer gene and the first tumor-suppressor gene to be cloned, and it has proven to be biologically important. The RB protein was shown to be a principal controller of the cell cycle in all human tissues. Furthermore, RB protein is inactivated by specific proteins produced by DNA tumor viruses, thereby demonstrating the oncogenic mode of action of their already known transforming factors. Showing this inactivation was, historically, critically important in resolving conflicting notions of viral versus genetic oncogenesis, just as the RNA tumor viruses directed investigators to proto-oncogenes.

## Lessons for Other Cancers

Pediatric oncologists treating retinoblastoma continue to address problems of general interest to all oncologists. The discovery that *RB1* is a gene whose mutations can also predispose a person to sarcomas, which in turn can be initiated by ionizing radiation, has implications for other cancers, and pediatrics has led the way in modifying therapy to reduce the probability of such a consequence. The use of chemotherapy and focal treatment to reduce the need for bilateral enucleation (surgical removal of the eye from its orbit) and/or radiation provides another example of the amelioration of late effects. The incidences of both late mortality and blindness have been reduced.

Retinoblastoma has been a model for guiding our understanding of cancer genes.

Following the cloning of *RB1*, more than 40 hereditary cancer genes have been cloned, including the gene for familial adenomatous polyposis, *APC*. As with retinoblastoma, *APC* was shown to be somatically mutant in a large majority of nonhereditary colon cancers, thereby stimulating interest in the study of hereditary cancer for the purpose of illuminating the biology of all cancer. However, the common carcinomas are often preceded by benign precursor lesions, such as the colonic polyp, whose diagnosis and removal can be an important preventive measure, especially since there is often a considerable time interval between formation of the precursor and its malignant transformation.

## Making Further Clinical Progress

Further clinical progress may depend on a deeper understanding of the mechanisms of transformation in cells with defective or absent RB protein. Still unanswered is the following question: why is *RB1* a retinoblastoma gene? Knowledge of control of the cell cycle by RB protein does not explain the protein's tissue specificity or its importance for retinal development. Following the cloning of *RB1*, knockout mice were produced, enabling the production and study of homozygotes. The homozygous state is lethal to embryos, with developmental defects in multiple tissues, including the brain. If heterozygous humans did not also develop tumors, we might know *RB1* as a recessive, developmental, lethal gene. Can the developmental defects be explained by what is known about control of the cell cycle by RB protein? Apparently not.

Now Dyer and colleagues, and other researchers [1] have discovered that RB protein interacts with other proteins that have separate developmental roles. A mutant retinal tissue progenitor cell is apparently arrested along its developmental pathway, and the arrested daughter cells continue to divide indefinitely. This discovery has stimulated a search for separate agents affecting either differentiation or development, one such combination being carboplatin and topotecan.

Even if a cure is usually accomplished with the application of current knowledge, there remain two problems. The first problem is the lethal progression of a few cases. The second is the problem of late effects, especially the difficult effect of second cancers in hereditary cases. Since these problems are also issues for other cancers, retinoblastoma assumes a special importance, with a seemingly better opportunity to identify secondary events in tumors. The rapid growth of retinoblastoma may facilitate recognition of rate-limiting steps in progression, especially with the availability of a good animal model. Since *RB1* and other functionally related genes are somatically mutant in so many cancers, study of them should be widely relevant.

## A New Role for Pediatric Oncologists?

Can pediatric oncologists participate directly in reducing the burden of cancer in adults? Is there a possibility of prevention? I have serious doubts that much could be done to prevent cancer in children, but intervention may be feasible in hereditary conditions that cause cancer in adults. Thus, both familial adenomatous polyposis and neurofibromatosis type 1 have incidences more than twice that of retinoblastoma. Successful intervention in childhood might possibly lead to delay or prevention of later malignancies. Any success would also be relevant to prevention of nonhereditary cancer, which would be especially important for colon cancer since most cases involve somatic mutations in the *APC* gene. Pediatric oncologists may find themselves in a new leadership role.
